# Information Spread and Topic Diffusion in Heterogeneous Information Networks

**DOI:** 10.1038/s41598-018-27385-2

**Published:** 2018-06-22

**Authors:** Soheila Molaei, Sama Babaei, Mostafa Salehi, Mahdi Jalili

**Affiliations:** 1 Faculty of New Sciences and Technologies, University of Tehran, Tehran, Iran; 2 School of Computer Science, Institute for Research in Fundamental Science (IPM), Tehran, Iran; 3 School of Engineering RMIT University, Melbourne, Australia

## Abstract

Diffusion of information in complex networks largely depends on the network structure. Recent studies have mainly addressed information diffusion in homogeneous networks where there is only a single type of nodes and edges. However, some real-world networks consist of heterogeneous types of nodes and edges. In this manuscript, we model information diffusion in heterogeneous information networks, and use interactions of different meta-paths to predict the diffusion process. A meta-path is a path between nodes across different layers of a heterogeneous network. As its most important feature the proposed method is capable of determining the influence of all meta-paths on the diffusion process. A conditional probability is used assuming interdependent relations between the nodes to calculate the activation probability of each node. As independent cascade models, we consider linear threshold and independent cascade models. Applying the proposed method on two real heterogeneous networks reveals its effectiveness and superior performance over state-of-the-art methods.

## Introduction

Many real systems can be modeled by networks where a number of individuals interact through a connection graph. Examples of networked systems include the Internet, World Wide Web, the human brain, power grids, online social networks, transportation and water distribution networks. Various dynamical phenomena have been studied on complex networks including synchronisation^1^, consensus^2^, opinion formation^3,4^ and information spread^5^. Network topology has the major role in how such dynamical processes evolve on networks. Certain topologies might facilitate synchronisation or information spread, while some other network structures might disrupt such activities^6,7^.

Information diffusion is one of the widely studied dynamical processed on networks, which has potential applications in fields. Information such as a news, innovation, virus or malware, starts from a set of seed nodes and propagates throughout the network. There is a rich literature on information diffusion on complex networks, where different models and their interplay with network topology have been studied^1^. Previous research works have mainly considered heterogeneous networks. An information network *G* = (*V*, *E*) with *V* as the set of nodes and *E* as the set of edges, is a homogeneous network if the edges and nodes are of the same type. Networks with nodes and/or edges from more than one type are called heterogeneous networks^8–10^. For example, in DBLP network, which is a major bibliography provider in computer science, the nodes are authors, papers, venues (journals/conferences). In this network, edges can be author-author relationship when they co-author a paper, or author-venue relationship when an author participates in a conference.

**Figure 1:**
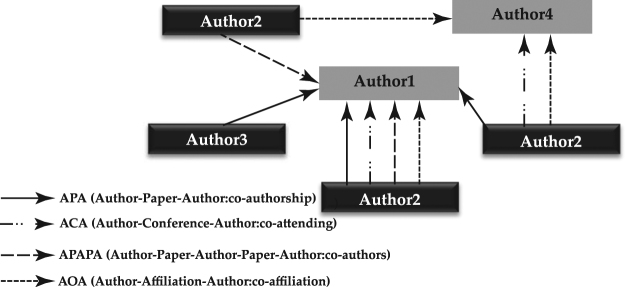
An Example of a heterogeneous network, where “Data mining” topic propagates along different types of relationships among authors. Black nodes are authors who have already pursued the topic, while gray nodes represent authors that may pursue the topic at the next timestamp.

Here we model information diffusion or more specifically topic diffusion in heterogeneous information networks. To this end, we use the concept of meta-path, which is defined in heterogeneous networks. A meta-path *P* is a path defined over the general schema of the network *T*_*G*_ = (*A*, *R*), where *A* and *R* denote the nodes and their relations, respectively. The meta-path is denoted by ${A}_{1}\mathop{\longrightarrow }\limits^{{R}_{1}}{A}_{2}\mathop{\longrightarrow }\limits^{{R}_{2}}\mathrm{...}\mathop{\longrightarrow }\limits^{{R}_{l}}{A}_{l+1}$, where *l* is an index indicating the corresponding meta-path. The aggregated relationship is obtained as *R* = *R*_1_*oR*_2_*o*...*R*_*l*_ between different types of nodes *A*_1_ to *A*_*l* + 1_, where o is the composition operator. For instance, in DBLP network, each of the author-paper-author and author-conference-author relations is considered to be an individual meta-path. Figure 1 is an example of “Data mining” topic propagation that authors can be connected to one another through different meta-paths in DBLP network.

Recently, much attention has been given to employing non-homogeneous networks in classification and ranking tasks. For instance, sentiment classification of product reviews using heterogeneous networks was addressed by Zhou *et al*.^11^. In this process, a heterogeneous network connects the users, products, and words, based on which the learning process is conducted using sentiment classification. In this regard, Zhou *et al*.^11^ proposed a co-ranking method which classifies the authors and documents separately based on random walks. Angelova *et al*.^12^ presented a new classification method for the DBLP heterogeneous network. Mining of heterogeneous networks was addressed in a number of studies^13–15^. For example, Boccaletti and others^16^ studied mining of homogeneous information networks through their decomposition into multiple homogeneous networks. The idea of citation recommendation using mining in heterogeneous networks was proposed by Liu *et al*.^17^. Heterogeneous networks have also been employed in healthcare. Some papers^18–20^ focused on epidemic spreading on heterogeneous networks. Considering an epidemic threshold, Wang and Dai^21^ addressed virus spreading in heterogeneous networks based on the well-known susceptible-infected-susceptible model. Moreover, it was shown by Yang *et al*.^22^ that by considering heterogeneity between people, a heterogeneous network is created which is resistant against epidemic spread of virus. Epidemic spreading is important issue that was considered in other networks likes time-varying networks^23^ and adaptive network^24^. Nadini *et al*.^23^ used SIR and SIS models and investigated effects of modular and temporal connectivity patterns on epidemic spreading.

Link prediction in heterogeneous networks has also been addressed. Shakibian and Moghadam Charkari^25^ used meta-paths for prediction and Jalili and Orouskhani^26^ formulated drug response prediction as a link prediction problem using kernelised Bayesian multitask learning algorithm. Some works have considered information diffusion on these networks. Sermpezis and his colleagues^27^ used degree distribution for the process of information diffusion assuming that diffusion takes place between two nodes at random times. Zhou and Liu^28^ presented a social influence based clustering framework has been presented for analyzing heterogeneous information networks. Moreover, a heterogeneous network model was proposed for new product diffusion in two stages by Li and Jin^29^; the first stage is transition of information concerning new products to customers through advertisement, and the second stage is changing customer priorities through persuasive advertisements.

As another definition, heterogeneous networks are referred to as multilayer networks, where the nodes and/or edges can be of different types. In many studies in this field, the concept of heterogeneous networks has been used to present a different definition for the infrastructure networks, based on which the concepts of diffusion are explained. Multilayer networks with all nodes from the same type are often called multiplex networks; a number of works have considered link prediction problem in multiplex networks^16,26^.

Some works have studied topic diffusion in heterogeneous networks. The concept of similarity based on meta-paths (known as Pathsim), between each two nodes was utilised and predictions were made by generalising the Linear threshold (LT) model by Gui and *et al*.^30^. Pathsim was considered as a weight between each two nodes in this method through which predictions were conducted^31,32^. In our proposed method, each meta-path instance is considered as a path by considering different meta-paths, and the conditional probability model is used to calculate the activation probability of each node. Also, two different diffusion models are used including Independent Cascade (IC) and LT. In these models, first all nodes are considered to be inactive. Then, an initial set of seed nodes are activated and LT/IC is used to activate the subsequent nodes. In IC model, an inactive node is activated under the influence of the active node with the highest probability of influence^33^. In this model, a probability is assigned to each active node for activating its neighbors; the probability of activation of node *w* triggered by node *v* is denoted as *P*(*v*|*w*). Every newly-activated node *v* attempts to trigger its inactive neighbors. If successfully triggered, node *w* is activated in the next step and triggers its inactive neighbors. Once a node is activated, it has a single chance to independently influence each of its neighbors. In LT mode, each inactive node is activated if the portion of its activated neighbors is more than a threshold *θ* ∈ [0, 1]^34^. Indeed, an inactive node is activated if and only if the total weight of all its activated neighbors exceeds a given threshold *θ*_*u*_, as equation (1).

1 $$\begin{array}{l}\sum _{v\in {\varepsilon }_{u}}{W}_{u,v} > \,={\theta }_{u}\end{array}$$

where *ε*_*u*_ is the active neighbors of node *u* and *W*_*u*,*v*_ represents the weight of the link between nodes *u* and *v*. Watts^35^ studied the role of threshold values and network structure in the information diffusion. Gui *et al*.^30^ proposed a model called Multi-Relational Linear Threshold Model - Relation Level Aggregation (MLTM-R), which studied how LT model behaves in heterogeneous networks. Our proposed model is compared to this model. The major contributions of this study are:

1. We propose two novel topic diffusion models in heterogeneous networks considering different meta-paths, meaning that the influence of each relation is individually learned.
2. The dependency of active nodes to inactive ones is considered and conditional probability is employed to obtain the possibility of activation of each inactive node.
3. Two frequently used models (LT and IC) are studied in heterogeneous networks and their behavior is compared in two real datasets. We show that IC model has more accurate answer than LT model in properly modeling topic diffusion in heterogeneous networks.

## Methods

This study incorporates conditional probability for calculating the activation probability of inactive nodes by neighboring active nodes. This is in fact known as information propagation probability which defines the probability that an active node activates an inactive neighbor. This propagation probability is calculated considering meta-paths and using Bayesian framework. It is assumed that inactive nodes are dependent on the active ones. IC and LT models are employed for the process of information distribution. The stages involved in the proposed method are briefly presented in algorithm 1 with every stage being explained separately in the following subsections.

### Datasets

A time-stamp of a year is defined for both datasets, based on which the training set and the test set are created as explained in the followings:

- **DBLP (computer science bibliography)**^36^: Objects indicate authors in this network. Different meta-path such as APA (Author-Paper-Author), ACA (Author-Conference-Author), APAPA (Author-Paper-Author-Paper-Author), and ACACA (Author- Conference -Author- Conference -Author) are considered. Different topics are extracted from this dataset, and information diffusion about a specific topic is investigated. This dataset include information from 1954 to 2016.
- **PubMed Dataset**^37,38^: In this network, the authors are represented by objects and meta-paths APA and APAPA are used. The dataset consists of information from 1950 to 2013. Information of both datasets is given in Table 1.

**Table 1:** Information of DBLP and PubMed datasets used in this work.

Dataset	Authors	Papers
DBLP	215222	105372
PubMed	1219686	459726

#### Evaluation criteria

All nodes with published papers on our particular topic of interest are tagged as active and the rest as inactive. Assuming the nodes to be predicted at time *t*, the training and test sets are considered as follows:

*Training set:* Those within the time period from *t* − 4 to *t* − 2 are considered as the training set.

*Test set:* Those within the time period from *t* *−* *1* to t are considered as the test set. Additionally, the nodes tagged as active up to the time *t* − 2 are considered as the seed nodes that are activated initially in the start of the diffusion process.

We use Precision and Recall, F-score, and Recall criteria to assess the performance. These metrics are defined as follows.

2 $$Precision=\frac{TF}{TF+FP},Recall=\frac{TP}{TP+FN},F-Score=\frac{2\ast (Precision\ast Recall)}{Precision+Recall}$$

where True Positive (TP) is the active nodes that are correctly tagged as active by the algorithm, True Negative (TN) is the inactive nodes that are correctly tagged as inactive by the algorithm, False Positive (FP) is the active nodes that are falsely tagged as inactive by the algorithm, and False Negative (FN) is the inactive nodes that are falsely tagged as active by the algorithm.

**Figure 2:**
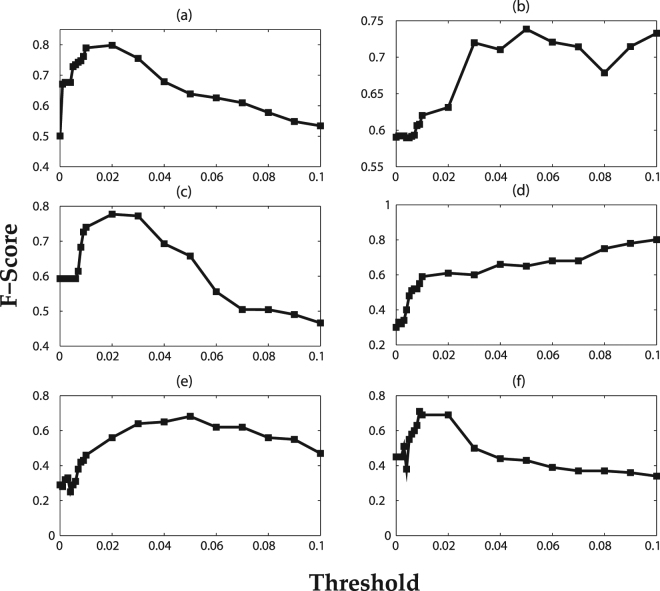
F-Score as a function of the threshold of LT model in DBLP dataset for selected topics. The figure shows F-score for topics (**a**) Data Mining, (**b**) Machine Learning, (**c**) Social Networks, (**d**) Healthcare, (**e**) DNA and (**f**) Infectious Disease. The optimal threshold for each topic is the one with the highest F-score.

In IC model, let *S*_*t*_ ⊆ *V* be the set of nodes that are activated at step *t* ≥ 0, with *S*_0_ = *S*. At step *t* + 1, every node *u* ∈ *S*_*t*_ may activate its out-neighbors *v* ∈ *V* with a propagation probability of *P*(*v*|*u*). One should also consider the activation threshold for LT model. We study how the diffusion process depends on the threshold value. Initially, the optimal threshold limit is required to be calculated from the training set in order to obtain the evaluation criteria according to the third step of the algorithm 1. Figure 2 shows the F-scores as a function of the threshold value when considering diffusion of the selected topics in DBLP dataset. As it is seen, one can often obtain an optimal value for the threshold for which the F-score is the highest. Note that F-score scales in the range [0, 1], where 1 indicates the best performance. This optimal threshold varies across different topics, which indicates that different topics have different propagation mechanisms in this dataset. The obtained optimal threshold value is then applied to the test set to assess the performance. We also use recall measure to obtain the optimal threshold and the results are similar to those obtained based on F-score (results not shown here).

### Calculating propagation probability of Nodes

Propagation probabilities for all edges and nodes are calculated in this stage. In order to calculate the activation probability of each node according to its neighboring nodes, the influence probabilities of each node and edge are calculated considering meta-paths.

*Edge Propagation Probability:* In heterogeneous networks, different routes are available for meta-paths. Hence, for every pair of nodes *v*_1_ and *v*_2_ in meta-path *k*, the edge probability is equal to the number of path instances between the two nodes divided by all the existing path instances between them, as shown in equation 3.

**Algorithm 1:**
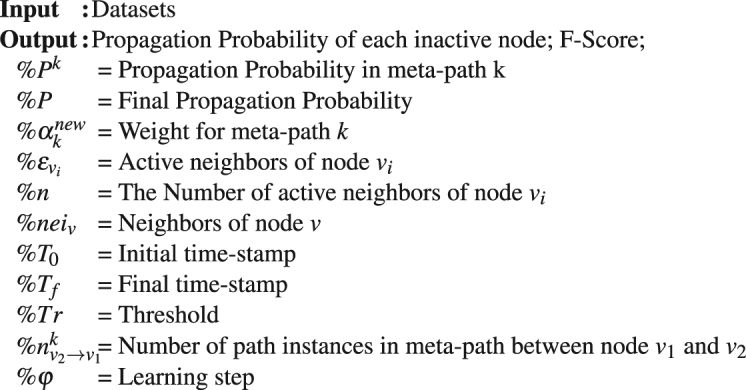
Heterogeneous Probability Model (HPM).

1. Calculate Probability for nodes:
  Find *P* for each pair of nodes *v*_1_ and *v*_2_
  $${P}^{k}({v}_{1}|{v}_{2})=\frac{{P}^{k}({v}_{1},{v}_{2})}{{P}^{k}({v}_{2})}=\frac{{n}_{{v}_{2}\to {v}_{1}}^{k}}{{\sum }_{r\in ne{i}_{v}}{n}_{{v}_{2}\to r}^{k}}$$
2. Create Propagation Flow Graph (PFG)
  For *t* from *T*_0_ to *T*_f_
    Insert edge from active nodes to inactive ones in the main graph
    Delete any edges between active nodes (there is no dependency between active nodes)
    Delete any edges between inactive nodes (there is no dependency between inactive nodes)
3. Calculate Propagation probabilities
  For t from *T*_0_ to *T*_f_
    For IC Model:
      For each inactive nodes do:
        Based on flow graph and ${{\alpha }}_{k}$- find $P({v}_{i}|\{{{\varepsilon }}_{{v}_{i}}\})$
        For *M* = 1 to *n*: calculate $P({v}_{i}|\{{{\varepsilon }}_{{v}_{{i}_{m}}}\})$
          $$P({v}_{i}|\{{{\varepsilon }}_{{v}_{{i}_{m}}}\})=\frac{{\sum }_{k=1}^{m}{{\alpha }}_{k}{n}_{{v}_{i}\to {{\varepsilon }}_{iM}}^{k}}{{\sum }_{k=1}^{m}{{\alpha }}_{k}\sum _{r\in ne{i}_{{{\varepsilon }}_{{v}_{iM}}}}{n}_{{{\varepsilon }}_{iM}\to r}^{k}}$$
        For *M* = 1 to *n*:
      If node(*v*_i_) was activated by one of active neighbors:
      Select max ($P({v}_{i}|{{\varepsilon }}_{v{i}_{M}})$) which activated *v*_*i*_ and consider *v*_*i*_ as active.else:
      Select max ($P({v}_{i}|{{\varepsilon }}_{v{i}_{M}})$) of neighbors and consider *v*_*i*_ as inactive.
      $$P({v}_{i}|{{\varepsilon }}_{{v}_{i}})=\mathop{{\rm{\max }}}\limits_{{\rm{M}}=\mathrm{1:}n}[P({v}_{i}|{{\varepsilon }}_{{v}_{{i}_{M}}})]$$
    For LT Model:
      For each inactive node do:
        Based on flow graph and ${{\alpha }}_{k}$ - find $P({v}_{i}|\{{{\varepsilon }}_{{v}_{i}}\})$
          $${P}^{k}({v}_{i}|\{{\varepsilon }_{{v}_{i}}\})=\frac{{P}^{k}({v}_{i},{\varepsilon }_{{v}_{{i}_{1}}},{\varepsilon }_{{v}_{{i}_{2}}},\ldots ,{\varepsilon }_{{v}_{{i}_{n}}})}{{P}^{k}(\{{\varepsilon }_{{v}_{i}}\})}$$
        If $P({v}_{i})\ge Tr$, consider *v*_*i*_ as active.
4. Learn ${{\alpha }}_{k}$ for each Meta-path *k*
  For *t* from *T*_0_ to *T*_*f*_:
  $${{\alpha }}_{k}^{new}={{\alpha }}_{k}^{new}+{\phi }\frac{{\partial }\,\mathrm{log}({P}({{U}}_{t}))}{{\partial }{{\alpha }}_{k}}$$
5. Calculate F-Score and Recall measures as equation (2)

3 $${P}^{k}({v}_{1},{v}_{2})=\frac{{n}_{{v}_{1}\to {v}_{2}}^{k}}{\sum _{i=1}^{{n}_{u}}\sum _{j=i}^{{n}_{u}}{n}_{{v}_{i}\to {v}_{j}}^{k}}$$

The above fraction can be considered as the information propagation probability between nodes *v*_1_ and *v*_2_. In equation (3), *P*^*k*^(*v*_1_, *v*_2_) denotes the probability of the edge between nodes *v*_1_ and *v*_2_ connecting in meta-path *k*. *n*_*u*_ is the total number of existing nodes and ${n}_{{v}_{1}\to {v}_{2}}^{k}$ represents the path instances between these nodes in meta-path *k*.

*Node Propagation Probability:* The strength of each node, i.e. the amount of information propagation the node is capable of, according to each meta-path is expressed by:

4 $${P}^{k}(v)=\frac{\sum _{r\in ne{i}_{v}}{n}_{v\to r}^{k}}{\sum _{i=1}^{{n}_{u}}\sum _{j=i}^{{n}_{u}}{n}_{{v}_{i}\to {v}_{j}}^{k}}$$

For instance, an author with a higher number of published papers should be assigned higher influence strength for information spread. Probability of propagation from node *v*_1_ to node *v*_2_ in meta-path *k* is expressed using equation (5).

5 $${P}^{k}({v}_{1}|{v}_{2})=\frac{{P}^{k}({v}_{1},{v}_{2})}{{P}^{k}({v}_{2})}=\frac{{n}_{{v}_{2}\to {v}_{1}}^{k}}{\sum _{r\in ne{i}_{v}}{n}_{{v}_{2}\to r}^{k}}$$

### Propagation flow graph

**Figure 3:**
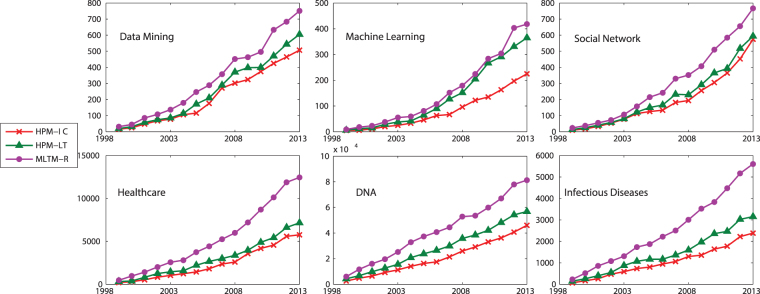
An illustrative example of Propagation Flow Graph where authors (active and inactive nodes) are connected to one another through papers (P). Edge of PFG illustrates that inactive nodes can be affected by active nodes.

The activation probability is assumed to be conditional as only an active node is capable of activating an inactive one, meaning that the direction of flow is always from the active node to the inactive one. Hence, the network is considered to be of Bayesian type. Additionally, we assume that active nodes are independent as an inactive node can only be activated by an active neighboring nodes and no flow may occur between two active nodes; hence no edge is considered between them. An implicit graph, with an example shown in Fig. 3, known as Propagation Flow Graph (PFG) is considered in this work. It should be noted that in order to calculate the node and edge propagation probabilities, the relationships between all nodes, both active or inactive ones are taken into account. In each state, if a node is activated, it is added to the PFG.

In our example shown in Fig. 3, nodes V2 and V4 are activate V1 as there are links from V2 and V4 to V1 on PFG. However, V3 can only be activated by V4 as there is no link from V1 to V3 on PFG. As V1 is activated in the first step, it can also affect V3 in the next step.

### Propagation Probability

In this section, the activation probability for each node is calculated according to IC and LT diffusion model.

#### IC Model

In IC model, each inactive node has a single change to be activated by one of its active neighbors. In other words, if an inactive node is not activated by a recently activated neighbor node, it will not be considered in the next steps for being activated. Here, among the neighboring nodes of an inactive node that activated this node, the one with the maximum probability is selected as the activating node. Otherwise, if the state of an inactive node does not change we select the maximum probability of neighbors as the probability of this inactive node. The propagation probability from active neighboring nodes (*ε*_*v*_) to an inactive node *v*_*i*_ through a given meta-path *k* is obtained according to:

6 $${P}^{k}({v}_{i}|\{{\varepsilon }_{{v}_{i}}\})=\mathop{{\rm{\max }}}\limits_{{\rm{M}}=\mathrm{1:}{\rm{n}}}[\frac{{P}^{k}({v}_{i},{\varepsilon }_{{v}_{{i}_{M}}})}{{P}^{k}({\varepsilon }_{{v}_{iM}})}]$$

As mentioned before, we assume that active nodes are independent since no flow may occur between two active nodes. Since the overall probability is obtained as the sum of meta-paths, the overall activation probability of node *v* can be obtained as:

7 $$P(v)=\sum _{k=1}^{m=number\,of\,metapaths}{\alpha }_{k}{P}^{k}(v)$$

which means that a coefficient *α*_*k*_ is assigned to each meta-path to obtain the overall probability. Among the active neighboring nodes of inactive node *v*_*i*_, the one with the maximum probability is selected as the activating node for node *v*_*i*_.

8 $$\begin{array}{ccc}\{{\varepsilon }_{{v}_{i}}\} & = & \{{\varepsilon }_{{v}_{{i}_{1}}},{\varepsilon }_{{v}_{{i}_{2}}},\ldots ,{\varepsilon }_{{v}_{{i}_{n}}}\}{\rm{\& }}{\varepsilon }_{{v}_{{i}_{1}}}\perp {\varepsilon }_{{v}_{{i}_{2}}}\perp \ldots \perp {\varepsilon }_{{v}_{{i}_{n}}}\\ P({v}_{i}|\{{\varepsilon }_{{v}_{i}}\}) & = & \mathop{max}\limits_{{\rm{M}}=1:{\rm{n}}}\,[\frac{{\sum }_{k=1}^{m}{\alpha }_{k}{P}^{k}({v}_{i},{\varepsilon }_{{v}_{{i}_{M}}})}{{\sum }_{k=1}^{m}{\alpha }_{k}{P}^{k}({\varepsilon }_{{v}_{{i}_{M}}})}]\\  & \approx  & \frac{\frac{1}{{\sum }_{i=1}^{{n}_{u}}{\sum }_{j=1}^{{n}_{u}}{n}_{{v}_{i}\to {v}_{j}}}{\sum }_{k=1}^{m}{\alpha }_{k}{n}_{{v}_{{\rm{i}}}\to {\varepsilon }_{{v}_{{i}_{M}}}}^{k}}{\frac{1}{{\sum }_{i=1}^{{n}_{u}}{\sum }_{j=1}^{{n}_{u}}{n}_{{v}_{i}\to {v}_{j}}}{\sum }_{k=1}^{m}{\alpha }_{k}{\sum }_{r\in ne{i}_{{\varepsilon }_{{v}_{{i}_{M}}}}}{n}_{{\varepsilon }_{{v}_{{i}_{M}}}}^{k}\to r}\\  & \approx  & \mathop{max}\limits_{{\rm{M}}=1:{\rm{n}}}\,[\frac{{\sum }_{k=1}^{m}{\alpha }_{k}{n}^{k}{v}_{{\rm{i}}}\to {\varepsilon }_{{v}_{{i}_{M}}}}{{\sum }_{k=1}^{m}{\alpha }_{k}{\sum }_{r\in ne{i}_{{\varepsilon }_{{v}_{{i}_{M}}}}}{n}_{{\varepsilon }_{{v}_{{i}_{M}}}}^{k}\to r}]\end{array}$$

#### LT Model

As a more intuitive and closer assumption to the real world, LT model assumes that a node is activated if at least certain percentage of its neighbors have already been activated. In DBLP network for example, this means that the total number of studied papers from different authors can influence the author to publish a paper on a particular topic. The general type of LT model is as equation (1). On the other hand, due to assuming the conditional probability, we can obtain the probability of each inactive node. In this section, we keep the properties of LT model and conditional probability together. In this case, calculations of propagation probability through active neighboring nodes of node *v*_*i*_ are as follows:

9 $$\begin{array}{ccc}{P}^{k}({v}_{i}|\{{\varepsilon }_{{v}_{i}}\}) & = & \frac{{P}^{k}({v}_{i},{\varepsilon }_{{v}_{{i}_{1}}},{\varepsilon }_{{v}_{{i}_{2}}},\ldots ,{\varepsilon }_{{v}_{{i}_{n}}})}{{P}^{k}(\{{\varepsilon }_{{v}_{i}}\})}\\  & = & {\textstyle \tfrac{{P}^{k}({v}_{i}|{\varepsilon }_{{v}_{{i}_{1}}})\times {P}^{k}({v}_{i}|{\varepsilon }_{{v}_{{i}_{2}}})\times \ldots \times {P}^{k}({v}_{i}|{\varepsilon }_{{v}_{{i}_{n}}})\times {P}^{k}({\varepsilon }_{{v}_{{i}_{1}}})\times {P}^{k}({\varepsilon }_{{v}_{{i}_{2}}})\times \ldots \times {P}^{k}({\varepsilon }_{{v}_{{i}_{n}}})}{{P}^{k}({\varepsilon }_{{v}_{{i}_{1}}})\times {P}^{k}({\varepsilon }_{{v}_{{i}_{2}}})\times \ldots \times {P}^{k}({\varepsilon }_{{v}_{{i}_{n}}})}}\\  & = & {P}^{k}({v}_{i}|{\varepsilon }_{{v}_{{i}_{1}}})\times {P}^{k}({v}_{i}|{\varepsilon }_{{v}_{{i}_{2}}})\times \ldots \times {P}^{k}({v}_{i}|{\varepsilon }_{{v}_{{i}_{n}}})=\prod _{q=1}^{n}{P}^{k}({v}_{i}|{\varepsilon }_{{v}_{{i}_{q}}})\end{array}$$

It is obvious that each node *v*_*i*_ should have more ${\prod }_{q=1}^{n}P({v}_{i}|{\varepsilon }_{{v}_{{i}_{q}}})$ for obtaining more influence. This means that with higher probability, the neighbors of node *v*_*i*_ will have more influence on it, which leads to:

10 $$\prod _{q=1}^{n}P({v}_{i}|{\varepsilon }_{{v}_{{i}_{q}}})\ge {\lambda }_{{v}_{i}}$$

We can infer that if multiplication of the neighbors’ probability of an inactive node *v*_*i*_ becomes more than the threshold ${\lambda }_{{v}_{i}}$, the inactive node is more likely to be activated. Let us multiply a constant value *υ* in both sides of equation 10 which does not change the final result:

11 $$\prod _{q=1}^{n}\upsilon P({v}_{i}|{\varepsilon }_{{v}_{{i}_{q}}})\ge {\upsilon }^{n}{\lambda }_{{v}_{i}}$$

By making logarithm from both sides of the above equation, we have equation (12) as:

12 $${\mathrm{log}}_{n}(\,\prod _{q=1}^{n}\upsilon P({v}_{i}|{\varepsilon }_{{v}_{{i}_{q}}})) > \,={\mathrm{log}}_{n}({\upsilon }^{n}{\lambda }_{{v}_{i}})\to \sum _{q=1}^{n}{\mathrm{log}}_{n}(\upsilon P({v}_{i}|{\varepsilon }_{{v}_{{i}_{q}}})) > \,={\theta }_{vi}\,\& \,{\mathrm{log}}_{n}(\upsilon P({v}_{i}|{\varepsilon }_{{v}_{{i}_{q}}})) < \,=1$$

Equation (12) shows that by considering *W*_*i*_ as ${log}_{n}(\upsilon P({v}_{i}|{\varepsilon }_{viq}))$ we kept the LT conditions and also we used conditional probability.

#### Learning model

Since information diffuses from active to inactive nodes, the flow of propagation is considered as a directed graph from active to inactive nodes. Moreover, due to their active state, no edge is considered between active nodes. Hence, according to PFG, the probability of all nodes is obtained through individual multiplication of active and inactive nodes. In the following, we explain the learning process used for IC and LT models.

### IC model

If *U*_*t*_ is the set of all graph nodes, *V*_*t*_ the set of active nodes and *R*_*t*_ the set of inactive nodes at time *t*, the propagation probability for nodes is obtained by:

13 $$P({U}_{t})=\prod _{t\in T}\prod _{v\in {V}_{t}}P(v)\prod _{r\in {R}_{t}}\mathrm{(1}-P(r|\{{\varepsilon }_{r}\}))$$

The objective is to maximise *P*(*U*_*t*_); the probability of active nodes (*P*(*V*_*t*_)) as well as that of unity minus the probability of inactive nodes (1 − *P*(*r*|{*ε*_*r*_})) should be maximised to obtain the best results. For convenience, the function can be converted to log-likelihood function as:

14 $$\begin{array}{rcl}log(P({U}_{t})) & = & \sum _{t\in T}[\sum _{v\in {V}_{t}}\sum _{k\mathrm{=1}}^{m}\mathrm{log}({\alpha }_{k}{P}^{k}(v))+\sum _{r\in {R}_{t}}(1-P(r|\{{\varepsilon }_{r}\}))]\\ 1-P(r|\{{\varepsilon }_{r}\}) & = & 1-\mathop{{\rm{\max }}}\limits_{J=\mathrm{1:}n}[\frac{{\sum }_{k=1}^{m}{\alpha }_{k}{P}^{k}(r,{{\varepsilon }}_{{r}_{J}})}{{\sum }_{k=1}^{m}{\alpha }_{k}{P}^{k}({{\varepsilon }}_{{r}_{J}})}]=\mathop{{\rm{\min }}}\limits_{{\rm{J}}=\mathrm{1:}{\rm{n}}}[\frac{{\sum }_{k=1}^{m}{\alpha }_{k}{P}^{k}({\varepsilon }_{{r}_{J}})-{\sum }_{k=1}^{m}{\alpha }_{k}{P}^{k}(r,{{\varepsilon }}_{{r}_{J}})}{{\sum }_{k=1}^{m}{\alpha }_{k}{P}^{k}({{\varepsilon }}_{{r}_{J}})}]\\ {\phi }\frac{\partial log(P({U}_{t}))}{\partial {\alpha }_{k}} & = & \sum _{t\in T}[\sum _{v\in {V}_{t}}\frac{P(v)}{{\sum }_{k=1}^{m}{\alpha }_{k}P(v)}+\sum _{r\in {R}_{t}}\mathop{{\rm{\min }}}\limits_{{\rm{J}}=\mathrm{1:}{\rm{n}}}[\begin{array}{l}\frac{{P}^{k}({{\varepsilon }}_{{r}_{J}})-{P}^{k}(r,{{\varepsilon }}_{{r}_{J}})}{{\sum }_{k=1}^{m}{\alpha }_{k}{P}^{k}({{\varepsilon }}_{{r}_{J}})-{\sum }_{k=1}^{m}{\alpha }_{k}{P}^{k}(r,{{\varepsilon }}_{{r}_{J}})}\\ -\frac{{P}^{k}({{\varepsilon }}_{{r}_{J}})}{{\sum }_{k=1}^{m}{\alpha }_{k}{P}^{k}({{\varepsilon }}_{{r}_{J}})}\end{array}]]\end{array}$$

### LT model

Similar to the log-likelihood function in IC model, this can also be obtained when using LT as influence model. The resulting equation is as follows:

15 $$\begin{array}{rcl}P({U}_{t}) & = & \prod _{t\in T}\prod _{v\in {V}_{t}}P(v)\mathop{\Pi }\limits_{r\in {R}_{t}}\mathrm{(1}-P(r|\{{\varepsilon }_{r}\}))\\ log(P({U}_{t})) & = & \sum _{t\in T}[\sum _{v\in {V}_{t}}\sum _{k=1}^{m}\mathrm{log}({\alpha }_{k}{P}^{k}(v))+\sum _{r\in {R}_{t}}(1-P(r|\{{\varepsilon }_{r}\}))]\\ {\phi }\frac{\partial log(P({U}_{t}))}{\partial {\alpha }_{k}} & = & \sum _{v\in {V}_{t}}\frac{P(v)}{{\sum }_{k=1}^{m}{\alpha }_{k}P(v)}+\sum _{r\in {R}_{t}}\varphi \frac{\partial }{\partial {\alpha }_{k}}log[1-\tfrac{{\sum }_{k=1}^{m}{\alpha }_{k}\{{P}^{k}({\rm{r}},{\varepsilon }_{{r}_{1}})\times {P}^{k}({\rm{r}},{\varepsilon }_{{r}_{2}})\times \ldots \times {P}^{k}({\rm{r}},{\varepsilon }_{{r}_{n}})\}}{{\sum }_{k=1}^{m}{\alpha }_{k}\{{P}^{k}({\varepsilon }_{{r}_{1}})\times {P}^{k}({\varepsilon }_{{r}_{2}})\times \ldots \times {P}^{k}({\varepsilon }_{{r}_{n}})\}}]\\  &  & \times \,\varphi \frac{\partial }{\partial {\alpha }_{k}}log[1-\tfrac{{\sum }_{k=1}^{m}{\alpha }_{k}\{{P}^{k}({\rm{r}},{\varepsilon }_{{r}_{1}})\times {P}^{k}({\rm{r}},{\varepsilon }_{{r}_{2}})\times \ldots \times {P}^{k}({\rm{r}},{\varepsilon }_{{r}_{n}})\}}{{\sum }_{k=1}^{m}{\alpha }_{k}\{{P}^{k}({\varepsilon }_{{r}_{1}})\times {P}^{k}({\varepsilon }_{{r}_{2}})\times \ldots \times {P}^{k}({\varepsilon }_{{r}_{n}})\}}]\\  & = & \tfrac{\{{P}^{k}({\varepsilon }_{{r}_{1}})\times {P}^{k}({\varepsilon }_{{r}_{2}})\times \ldots \times {P}^{k}({\varepsilon }_{{r}_{n}})\}-\{{P}^{k}({\rm{r}},{\varepsilon }_{{v}_{1}})\times {P}^{k}({\rm{r}},{\varepsilon }_{{v}_{2}})\times \ldots \times {P}^{k}(r,{\varepsilon }_{{v}_{n}})\}}{{\sum }_{k=1}^{m}{\alpha }_{k}\{{P}^{k}({\varepsilon }_{{r}_{1}})\times {P}^{k}({\varepsilon }_{{r}_{2}})\times \ldots \times {P}^{k}({\varepsilon }_{{r}_{n}})\}-{\sum }_{k=1}^{m}{\alpha }_{k}\{{P}^{k}({\rm{r}},{\varepsilon }_{{r}_{1}})\times {P}^{k}(r,{\varepsilon }_{{r}_{2}})\times \ldots \times {P}^{k}(r,{\varepsilon }_{{r}_{n}})\}}\\  &  & -\,\tfrac{\{{P}^{k}({\varepsilon }_{{r}_{1}})\times {P}^{k}({\varepsilon }_{{r}_{2}})\times \ldots \times {P}^{k}({\varepsilon }_{{r}_{n}})\}}{{\sum }_{k=1}^{m}{\alpha }_{k}\{{P}^{k}({\varepsilon }_{{r}_{1}})\times {P}^{k}({\varepsilon }_{{r}_{2}})\times \ldots \times {P}^{k}({\varepsilon }_{{r}_{n}})\}}\end{array}$$

Ultimately, both models use equation (16) for calculating the coefficient of each meta-path (*α*_*k*_).

16 $${\alpha }_{k}^{new}={\alpha }_{k}^{new}+\frac{\partial log(P({U}_{t}))}{\partial {\alpha }_{k}}\& \sum _{k\in Metapaths}{\alpha }_{k}=1$$

### Example

In this section, we provide the above analysis on a sample network shown in Fig. 3. In this network, nodes *V*2 and *V*4 are active nodes, and thus can influence the inactive nodes *V*1, *V*3, *V*5 and *V*6, and activate them. Considering two meta-paths APA and APAPA, the probability of activation for each node can be calculated as follows:

$$\begin{array}{c}APA{:}\\ {P}^{APA}({v}_{1}|{v}_{2})=\frac{{n}_{{v}_{2}\to {v}_{1}}^{APA}}{{\sum }_{r\in ne{i}_{{v}_{2}}}{n}_{{v}_{2}\to {v}_{r}}^{APA}}=\frac{1}{2},{P}^{APA}({v}_{3}|{v}_{4})\\ \,\,\,\,\,\,\,=\,\frac{{n}_{{v}_{4}\to {v}_{3}}^{APA}}{{\sum }_{r\in ne{i}_{{v}_{4}}}{n}_{{v}_{4}\to {v}_{r}}^{APA}}=\frac{1}{8},{P}^{APA}({v}_{1}|{v}_{4})=\frac{{n}_{{v}_{4}\to {v}_{1}}^{APA}}{{\sum }_{r\in ne{i}_{{v}_{4}}}{n}_{{v}_{4}\to {v}_{r}}^{APA}}=\frac{1}{8},\\ {P}^{APA}({v}_{6}|{v}_{4})=\frac{{n}_{{v}_{4}\to {v}_{6}}^{APA}}{{\sum }_{r\in ne{i}_{{v}_{4}}}{n}_{{v}_{4}\to {v}_{r}}^{APA}}=\frac{1}{8},{P}^{APA}({v}_{5}|{v}_{4})=\frac{{n}_{{v}_{4}\to {v}_{5}}^{APA}}{{\sum }_{r\in ne{i}_{{v}_{4}}}{n}_{{v}_{4}\to {v}_{r}}^{APA}}=\frac{1}{4}\end{array}$$

$$\begin{array}{c}APAPA:\\ {P}^{APAPA}({v}_{1}|{v}_{2})=\frac{{n}_{{v}_{2}\to {v}_{1}}^{APAPA}}{{\sum }_{r\in ne{i}_{{v}_{2}}}{n}_{{v}_{2}\to {v}_{r}}^{APAPA}}=\frac{1}{8},{P}^{APAPA}({v}_{3}|{v}_{4})\\ \,\,\,\,\,\,\,=\,\frac{{n}_{{v}_{4}\to {v}_{3}}^{APAPA}}{{\sum }_{r\in ne{i}_{v}}{n}_{{v}_{4}\to {v}_{r}}^{APAPA}}=\frac{2}{25},{P}^{APAPA}({v}_{1}|{v}_{4})=\frac{{n}_{{v}_{4}\to {v}_{1}}^{APAPA}}{{\sum }_{r\in ne{i}_{{v}_{4}}}{n}_{{v}_{4}\to {v}_{r}}^{APAPA}}=\frac{11}{25},\\ {P}^{APAPA}({v}_{6}|{v}_{4})=\frac{{n}_{{v}_{4}\to {v}_{6}}^{APAPA}}{{\sum }_{r\in ne{i}_{{v}_{4}}}{n}_{{v}_{4}\to {v}_{r}}^{APAPA}}=\frac{6}{25},{P}^{APAPA}({v}_{5}|{v}_{4})=\frac{{n}_{{v}_{4}\to {v}_{5}}^{APAPA}}{{\sum }_{r\in ne{i}_{{v}_{4}}}{n}_{{v}_{4}\to {v}_{r}}^{APAPA}}=\frac{3}{25}\end{array}$$

where the values of *α*_*APA*_ and *α*_*APAPA*_ are learned for the corresponding meta-paths. Assuming the learned values for *α*_*APA*_ and *α*_*APAPA*_ as 0.6 and 0.4, respectively, the final activation probability is obtained as:

$$\begin{array}{c}{\rm{I}}{\rm{C}}:\\ \begin{array}{lll}P({v}_{1}|\{{v}_{2},{v}_{4}\}) & = & \mathop{{\rm{m}}{\rm{a}}{\rm{x}}}\limits_{{\rm{M}}=1:{\rm{n}}}\,[\frac{{\sum }_{k=1}^{m}{\alpha }_{k}{n}_{{v}_{1}\to {v}_{2}}^{k}}{{\sum }_{k=1}^{m}{\alpha }_{k}{\sum }_{r\in ne{i}_{{v}_{2}}}{n}_{{v}_{2}\to r}^{k}},\frac{{\sum }_{k=1}^{m}{\alpha }_{k}{n}_{{v}_{1}\to {v}_{4}}^{k}}{{\sum }_{k=1}^{m}{\alpha }_{k}{\sum }_{r\in ne{i}_{{v}_{4}}}{n}_{{v}_{4}\to r}^{k}}]\\  & = & \mathop{{\rm{m}}{\rm{a}}{\rm{x}}}\limits_{{\rm{M}}=1:{\rm{n}}}\,[\frac{0.6\times 3+0.4\times 3}{0.6\times 6+0.4\times 24},\frac{0.6\times 1+0.4\times 11}{0.6\times 8+0.4\times 25}]\\ P({v}_{3}|\{{v}_{4}\}) & = & \frac{0.6\times 1+0.4\times 2}{0.6\times 8+0.4\times 25},\\ P({v}_{5}|\{{v}_{4}\}) & = & \frac{0.6\times 2+0.4\times 3}{0.6\times 8+0.4\times 25},P({v}_{6}|\{{v}_{4}\})=\frac{0.6\times 1+0.4\times 6}{0.6\times 8+0.4\times 25}\end{array}\end{array}$$

$$\begin{array}{ccc}{\rm{L}}{\rm{T}}: &  & \\ {P}^{APA}({v}_{1}|\{{v}_{2},{v}_{4}\})=\frac{1}{2}\times \frac{1}{8}=\frac{1}{16},\\ {P}^{APAPA}({v}_{1}|\{{v}_{2},{v}_{4}\})=\frac{1}{8}\times \frac{11}{25}=\frac{11}{200}\end{array}$$

## Experimental Results

As mentioned for DBLP example, the authors are connected to one another according to the specified meta-path. For topic diffusion in such a graph, initially we need to select a special topic like “data mining”. The authors with papers related to the selected topic are considered as active nodes. These authors affect their neighbors in a way that of an inactive node (author) might be encouraged to write a paper in this filed affected by active author(s). If a neighbor writes paper in this field, they will be active and will then affect their neighbors.

**Figure 4:**
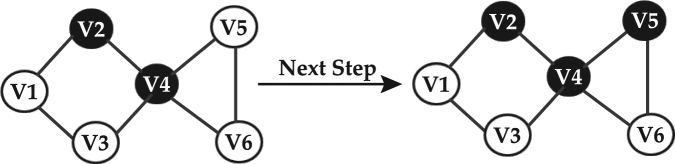
Process of activating inactive nodes affected by active neighbors for “Data Mining” topic.

Figure 4 shows an example in which in the first step nodes *V*2 and *V*4 are activated by “data mining” topic. Node *V*2 can activate node *V*1 while node *V*4 can activate nodes *V*3, *V*5 and *V*6. In this example, node *V*4 activates node *V*5, hence node *V*5 is persuaded to write paper in “data mining” topic. In this section, we apply the proposed model on two real datasets and discuss the results. We consider two popular datasets, DBLP and PubMed, which include information on authors, papers and venues. We also consider some topics including data mining, machine learning, social networks, healthcare, DNA, and infectious disease, for which the diffusion process is modeled. The topic selection is mainly due to their convenient frequency in the datasets and the considerable amount of data available for comparison and conclusion.

### DBLP

**Figure 5:**
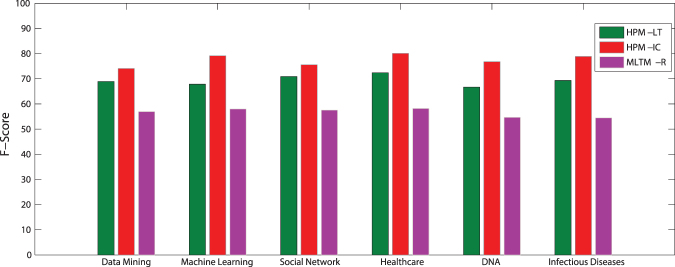
F-Score of the proposed method (HPM) with IC and LT as diffusion models (HPM-IC and HPM-LT) and the state-of-the-art method (MLTM-R) on DBLP dataset.

**Figure 6:**
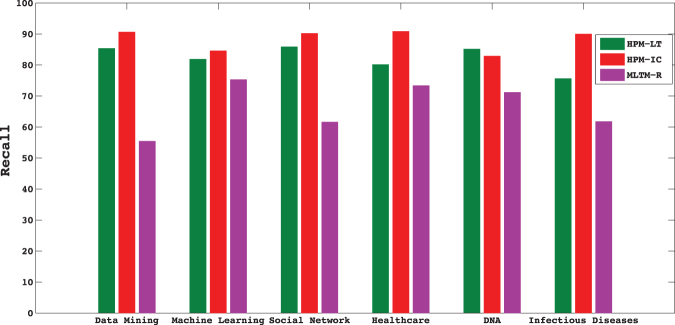
Recall of the methods on DBLP dataset.

In this dataset, information diffusion is investigated on the selected topics. The results of the proposed method is compared to the state-of-the-art method introduced by Gui *et al*.^30^, known as MLTM-R. Figures 5 and 6 compare the performance of the proposed model, Heterogeneous Probability Model (HPM), with MLTM-R in terms of F-score and Recall, respectively. Note that the original MLTM-R method is based on LT model for diffusion, while HPM works for both LT and IC models. As it can be seen, HPM significantly outperforms MLTM-R by providing much better F-score and Recall when IC model is used. An improvement of about 30–50% is obtained in HPM as compared to MLTM-R. Furthermore, these results show that one can obtain much better performance when IC model is used rather than LT. This indicates that IC model is better capable of modeling topic diffusion in this dataset.

**Figure 7:**
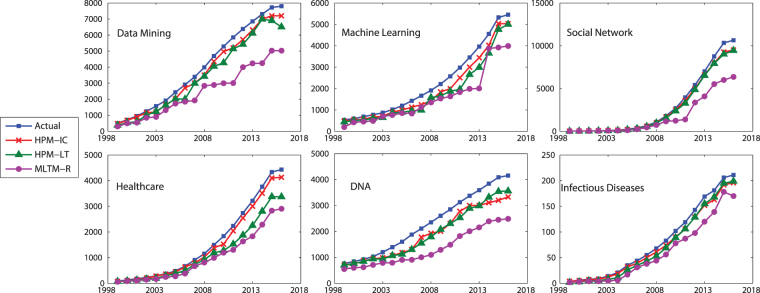
The number of correctly predicted active authors (TP) for the selected topics on DBLP dataset.

**Figure 8:**
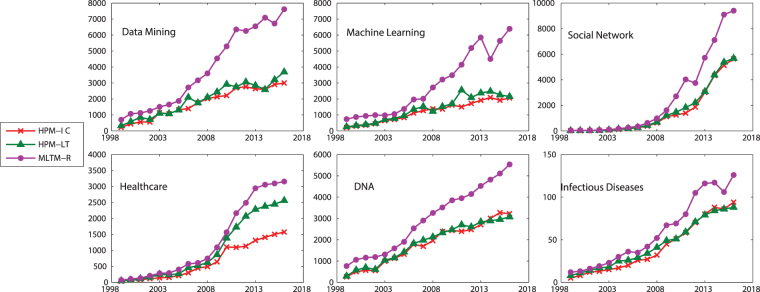
The number of authors who have been tagged incorrectly as active (FP) or inactive (FN) for the selected topics on DBLP dataset.

Figure 7 compares TP, i.e., the number of correctly predicted active authors, of the methods. It also include the actual TPs for different years, where the closer is the predicted value to these actual values, the better is the performance of the method. As it is seen, the proposed method with IC model (HPM-IC) has the closest predicted values to the actual ones, followed by HPM-LT and then MLTM-R. This performance is observed across all the selected topics and all years. Figure 8 shows the number of authors who have been incorrectly identified as active or inactive, where HPM-IC has the lowest values (i.e., the best performance) while MLTM has the worst performance.

#### PubMed

**Figure 9:**
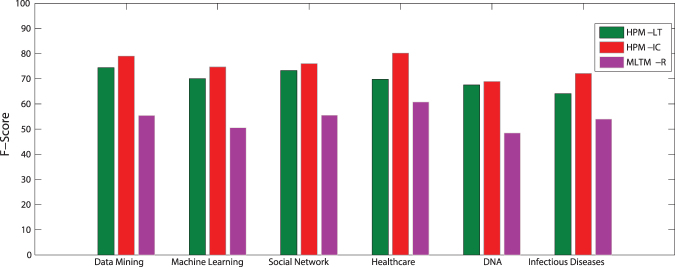
F-Score of the proposed method (HPM) with IC and LT as diffusion models (HPM-IC and HPM-LT) and the state-of-the-art method (MLTM-R) on PubMed dataset.

**Figure 10:**
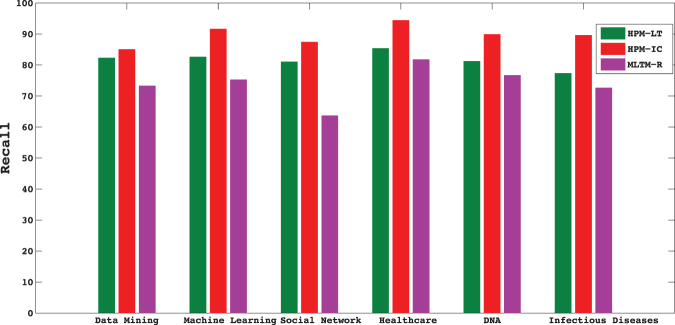
Recall of the methods on PubMed dataset.

**Figure 11:**
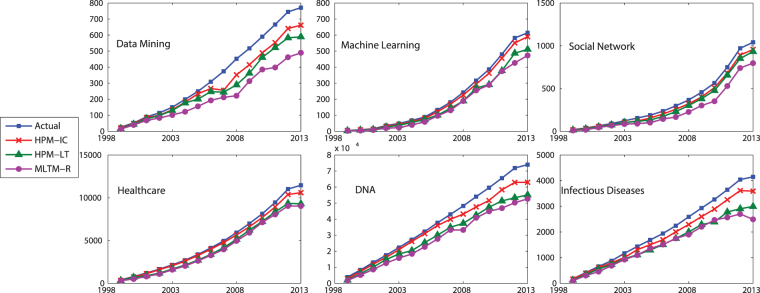
The number of correctly predicted active authors (TP) for the selected topics on PubMed dataset.

**Figure 12:**
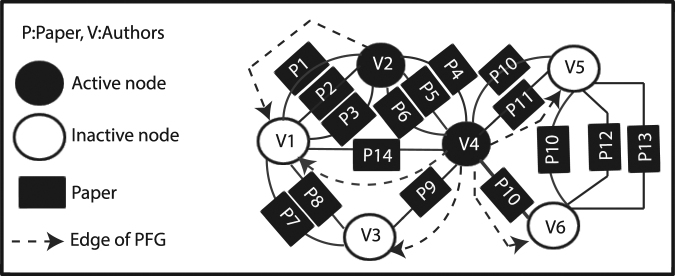
The number of authors who have been tagged incorrectly as active (FP) or inactive (FN) for the selected topics on PubMed dataset.

We apply the methods on PubMed dataset with the same selected topics. Figures 9 and 10 show the F-score and Recall of the methods, respectively. Similar to the other dataset, HPM significantly outperforms MLTM-R in all topics. Also, HPM-IC performs better HPM-LT. Figures 11 and 12 show the correctly identified active authors (TP) and incorrectly identified active and inactive authors (FN and FP), respectively. As it is seen, similar to the other dataset, HPM-IC has the best performance.

#### Analysis

Compared to MLTM-R method^30^, HPM-LT and HPM-IC methods significantly improve the F-score and Recall of the prediction, which is mainly due to the following reasons. MLTM-R uses pathsim to calculate the weight of each edge. Pathsim is not accurate in some cases^31,32^, as it does not obtain similarity value (or obtain low similarity scores) between two similar nodes in certain circumstances. However, in our proposed method, each meta-path instance is considered as a path by considering different routes between nodes, which eliminates the problems of Pathsim as there is no need to calculate the similarity for weights. The proposed method instead uses the conditional probability model to calculate the activation probability of each node. The inactive nodes are considered to be dependent on the active neighboring nodes. This is a realistic scenario as if an author decides to write a paper about an issue, they should have already be aware of the existence papers written by others (active nodes). Unlike the other method, in the proposed algorithm we separately consider the node and edge influence. The node influence is considered by having IC and LT models in which activation of inactive nodes is based on neighboring active nodes. The edge influence is considered as the extent to which the relation between two nodes is important for diffusion process i.e. a relation is more impressive if larger number of multipaths are found between two nodes. Topological properties of networks have significant influence on the way information propagates on them. DBLP has larger average degree than PubMed and having more connections facilitates spread. Our results also confirms this as the performance of the methods is better for DBLP than PubMed.

Better performance of the proposed strategy over of the previous model is due to considering information extracted from meta-paths. A meta-path is a path between any two nodes from different layers of an heterogeneous network. As meta-path traverses between different type of object, it can extract useful information on the structure of the network. method based on meta- paths have already been used for network analysis such as link prediction^5^. Our experiments shows that meta-paths are also important in the way information spread across layers and different object types. We also consider importance of the nodes by taking into account the paths passing through them (equation 4).

Our proposed method use meta-paths with different lengths. Two non-adjacent nodes of the same type, e.g. two authors in DBLP example, might be connected through meta-paths of length two or three. For example, in DBLP network when two authors who do not have any co-authored papers, both have papers with another authors, there is a meta-path of length two between these two authors. Considering such meta-paths allows one to account for such indirect connections between the nodes and taking into account the cross-layer information at the same time.

## Conclusion

This paper studied information spread and diffusion of scientific topics in heterogeneous networks. To this end, a novel method called HPM, was developed based on meta-paths and conditional probability. Moreover, propagation flow graph was defined to illustrate the diffusion flow from active to inactive nodes. Propagation probability was then calculated based on this graph and the coefficients of meta-paths were learned using the log-likelihood function. We considered two well-known diffusion models: Linear Threshold (LT) and Independent Cascade (IC) models. In LT model, inactive nodes are activated if the portion of their active neighbors is higher than a certain threshold. In IC model, the recently activate nodes activates its inactive neighbors with a certain probability. We considered the problem of topic diffusion in two real-world networks: DBPL and PubMed. The performance of the proposed model was compared with a state-of-the-art method, where our experimental results showed that the proposed method significantly outperform the other one. Also, Using IC as the diffusion model led to better performance than LT model.

## Electronic supplementary material

LaTeX Supplementary File

LaTeX Supplementary File

LaTeX Supplementary File

LaTeX Supplementary File

## References

[1] Arenas A, Díaz-Guilera A, Kurths J, Moreno Y, Zhou C (2008). Synchronization in complex networks. Physics Reports.

[2] Olfati-Saber R, Fax JA, Murray RM (2007). Consensus and Cooperation in Networked Multi-Agent Systems. Proceedings of the IEEE.

[3] Jalili M (2013). Social power and opinion formation in complex networks. Physica A: Statistical Mechanics and its Applications.

[4] Jalili M (2013). Effects of leaders and social power on opinion formation in complex networks. Simulation.

[5] Jalili, M. & Perc, M. Information cascades in complex networks. *Journal of Complex Networks*, 10.1093/comnet/cnx019 (2017).

[6] Kirst C, Timme M, Battaglia D (2016). Dynamic information routing in complex networks. Nature Communications.

[7] Zhang Z-K (2016). Dynamics of information diffusion and its applications on complex networks. Physics Reports.

[8] Deng, H., Han, J., Zhao, B., Yu, Y. & Lin, C. X. Probabilistic Topic Models with Biased Propagation on Heterogeneous Information Networks. *Kdd* 1271–1279, 10.1145/2020408.2020600 (2011).

[9] Han, J. Mining heterogeneous information networks by exploring the power of links. In *Discovery Science*, 13–30, 10.1007/978-3-642-04747-3_2 (2009).

[10] Sun Y, Han J (2013). Mining heterogeneous information networks: a structural analysis approach. ACM SIGKDD Explorations Newsletter.

[11] Zhou, D., Orshanskiy, S. A., Zha, H. & Giles, C. L. Co-ranking Authors and Documents in a Heterogeneous Network. In *Seventh IEEE International Conference on Data Mining (ICDM 2007)*, 739–744, 10.1109/ICDM.2007.57 (2007).

[12] Angelova R, Kasneci G, Weikum G (2012). Graffiti: graph-based classification in heterogeneous networks. World Wide Web.

[13] Sun Y, Han J (2012). Mining Heterogeneous Information Networks: Principles and Methodologies. Synthesis Lectures on Data Mining and Knowledge Discovery.

[14] Sun Y, Han J (2013). Mining heterogeneous information networks. ACM SIGKDD Explorations Newsletter.

[15] Kralj, J., Robnik-Šikonja, M. & Lavrač N. HINMINE: heterogeneous information network mining with information retrieval heuristics. *Journal of Intelligent Information Systems*, 10.1007/s10844-017-0444-9 (2017).

[16] Boccaletti S (2014). The structure and dynamics of multilayer networks. Physics Reports.

[17] Liu, X., Yingying, Yu, Guo, C., Sun, Y. & Gao, L. Full-text based context-rich heterogeneous network mining approach for citation recommendation. In *IEEE/ACM Joint Conference on Digital Libraries*, 361–370, 10.1109/JCDL.2014.6970191 (2014).

[18] Yang R (2007). Epidemic spreading on heterogeneous networks with identical infectivity. Physics Letters A.

[19] Moreno Y, Pastor-Satorras R, Vespignani A (2002). Epidemic outbreaks in complex heterogeneous networks. The European Physical Journal B.

[20] Salehi M (2015). Spreading processes in multilayer networks. IEEE Transactions on Network Science and Engineering.

[21] Wang L, Dai GZ (2008). Global stability of virus spreading in complex heterogeneous networks. Siam Journal on Applied Mathematics.

[22] Yang H, Tang M, Gross T (2015). Large epidemic thresholds emerge in heterogeneous networks of heterogeneous nodes. Scientific Reports.

[23] Nadini M (2018). Epidemic spreading in modular time-varying networks. Scientific Reports.

[24] Demirel G, Barter E, Gross T (2017). Dynamics of epidemic diseases on a growing adaptive network. Scientific reports.

[25] Shakibian H, Moghadam Charkari N (2017). Mutual information model for link prediction in heterogeneous complex networks. Scientific Reports.

[26] Jalili M, Orouskhani Y, Asgari M, Alipourfard N, Perc M (2017). Link prediction in multiplex online social networks. Royal Society Open Science.

[27] Sermpezis, P. & Spyropoulos, T. Information diffusion in heterogeneous networks: The configuration model approach. In *Proceedings - IEEE INFOCOM*, 3261–3266, 10.1109/INFCOM.2013.6567148 (2013).

[28] Zhou, Y. & Liu, L. Social influence based clustering of heterogeneous information networks. In *Proceedings of the 19th ACM SIGKDD international conference on Knowledge discovery and data mining*, 338–346, 10.1145/2487575.2487640 (2013).

[29] Li S, Jin Z (2014). Modeling and Analysis of New Products Diffusion on Heterogeneous Networks. Journal of Applied Mathematics.

[30] Gui, H., Sun, Y., Han, J. & Brova, G. Modeling Topic Diffusion in Multi-Relational Bibliographic Information Networks. In *Proceedings of the 23rd ACM International Conference on Conference on Information and Knowledge Management - CIKM ‘14*, 649–658, New York, New York, USA), 10.1145/2661829.2662000 (2014).

[31] Shang, J. *et al*. Meta-path guided embedding for similarity search in large-scale heterogeneous information networks. *preprint at* https://arxiv.org/abs/1610.09769 (2016).

[32] Kuck J, Zhuang H, Yan X, Cam H, Han J (2015). Query-Based Outlier Detection in Heterogeneous Information Networks. Advances in database technology: proceedings. International Conference on Extending Database Technology.

[33] Goldenberg J, Libai B, Muller E (2001). Talk of the network: A complex systems look at the underlying process of word-of-mouth. Marketing letters.

[34] Granovetter MS (1978). Threshold Models of Collective Behavior. American Journal of Sociology.

[35] Watts DJ (2002). A simple model of global cascades on random networks. Proceedings of the National Academy of Sciences.

[36] Tang, J. *et al*. Arnetminer: extraction and mining of academic social networks. In *Proceedings of the 14th ACM SIGKDD international conference on Knowledge discovery and data mining*, 990–998, 10.1145/1401890.1402008 (2008).

[37] Light RP, Polley DE, Börner K (2014). Open data and open code for big science of science studies. Scientometrics.

[38] LaRowe G, Ambre S, Burgoon J, Ke W, Börner K (2008). The scholarly database and its utility for scientometrics research. Scientometrics.

